# The relationship between Lipocalin-2 level and hepatic steatosis in obese patients with NAFLD after bariatric surgery

**DOI:** 10.1186/s12944-022-01622-0

**Published:** 2022-01-16

**Authors:** Jiaqi Chen, Shihui Lei, Yueye Huang, Xiaojuan Zha, Lei Gu, Donglei Zhou, Jun Li, Feng Liu, Nannan Li, Lei Du, Xiu Huang, Ziwei Lin, Le Bu, Shen Qu

**Affiliations:** 1 Department of Endocrinology and Metabolism, Shanghai Tenth People’s Hospital, Tongji University School of Medicine, 200072 Shanghai, China; 2 Department of Endocrinology and Metabolism, Suzhou Municipal Hospital, The Affiliated Suzhou Hospital of Nanjing Medical University, Suzhou, China; 3 Department of Gastrointestinal Surgery, Shanghai Tenth People’s Hospital, Tongji University School of Medicine, Shanghai, China; 4 Department of Gastroenterology, Shanghai Tenth People’s Hospital, Tongji University School of Medicine, Shanghai, China

**Keywords:** Lipocalin-2, Nonalcoholic fatty liver disease, Hepatic steatosis, Laparoscopic sleeve gastrectomy

## Abstract

**Background:**

Lipocalin-2 (LCN2) has a critical effect on obesity as well as its associated comorbidities. The present study focused on analyzing serum LCN2 levels of obese patients with nonalcoholic fatty liver disease (NAFLD) and on determining relationship of hepatic steatosis improvement with LCN2 levels after laparoscopic sleeve gastrectomy (LSG).

**Methods:**

This work enrolled ninety patients with obesity and NAFLD. Twenty-three of them underwent LSG. Anthropometric and biochemical parameters and serum LCN2 levels were determined at baseline and those at 6-month post-LSG. Controlled attenuation parameter (CAP) measured by FibroScan was adopted for evaluating hepatic steatosis.

**Results:**

Among severe obesity patients, serum LCN2 levels were significantly increased (111.59 ± 51.16 ng/mL vs. 92.68 ± 32.68 ng/mL, *P* = 0.035). The CAP value was higher indicating higher liver fat content (360.51 ± 45.14 dB/m vs. 340.78 ± 45.02 dB/m, *P* = 0.044). With regard to surgical patients, liver function, glucose, and lipid levels were significantly improved after surgery. Serum LCN2 levels significantly decreased (119.74 ± 36.15 ng/mL vs. 87.38 ± 51.65 ng/mL, *P* = 0.001). Decreased CAP indicated a significant decrease in liver fat content (358.48 ± 46.13 dB/m vs. 260.83 ± 69.64 dB/m, *P* < 0.001). The decrease in LCN2 levels was significantly related to the reduced hepatic fat content and improvement in steatosis grade after adjusting for gender, age, and BMI decrease.

**Conclusions:**

Serum LCN2 levels are related to obesity and NAFLD. The decreased serum LCN2 levels could be an indicator of hepatic steatosis improvement.

## Introduction

Nonalcoholic fatty liver disease (NAFLD) is featured by ectopic hepatic fat deposition. It is now becoming an urgent health problem worldwide [1, 2]. Its epidemiology is usually associated with obesity. Being overweight and obese in early life is an independent risk of NAFLD in adulthood [3]. As the mechanisms of NAFLD have not been fully clarified, there are no specific pharmacological interventions approved for its treatment. Targeting obesity is still the priority for the treatment of NAFLD [4].

Bariatric surgery is now recommended as an effective approach to treat clinically severe obesity or obesity with complications. In addition to the dramatic weight loss, improvements in hepatic steatosis and inflammation are observed after bariatric surgery. The regression of fibrosis and decreased incidence of HCC are also benefits of bariatric surgery [5]. These beneficial effects are largely attributed to metabolic improvement accompanied by weight loss.

Lipocalin-2 (LCN2) is a 25 kD secretory glycoprotein, which belongs to lipocalin transport protein family. It is encoded by the *Lcn2* gene located on chromosome locus 9q34.11 in human. LCN2 was first considered to function in the innate immune response because of its up-regulation during bacterial infection. By binding to and sequestering iron-containing siderophores LCN2 can prevent bacteria iron uptake and alleviate bacterial infection [6]. Subsequent studies demonstrated that LCN2 was associated with various disorders, such as obesity [7]. Soon afterward, interest was attracted to the relationship between LCN2 and metabolic disorders, as LCN2 has a critical effect on lipid metabolism and insulin resistance [7]. Several studies focused on LCN2 levels in NAFLD [8, 9]. However, the results from different studies were inconsistent and the relationship between LCN2 levels and hepatic steatosis is still unclear.

Thus, in the current study, the authors examined serum LCN2 levels in obese patients with NAFLD as well as its change in a subgroup of patients after laparoscopic sleeve gastrectomy (LSG). The authors also investigated the relationship between alterations in LCN2 levels and hepatic steatosis improvement after LSG.

## Methods

### Study design and patients

**Fig. 1:**
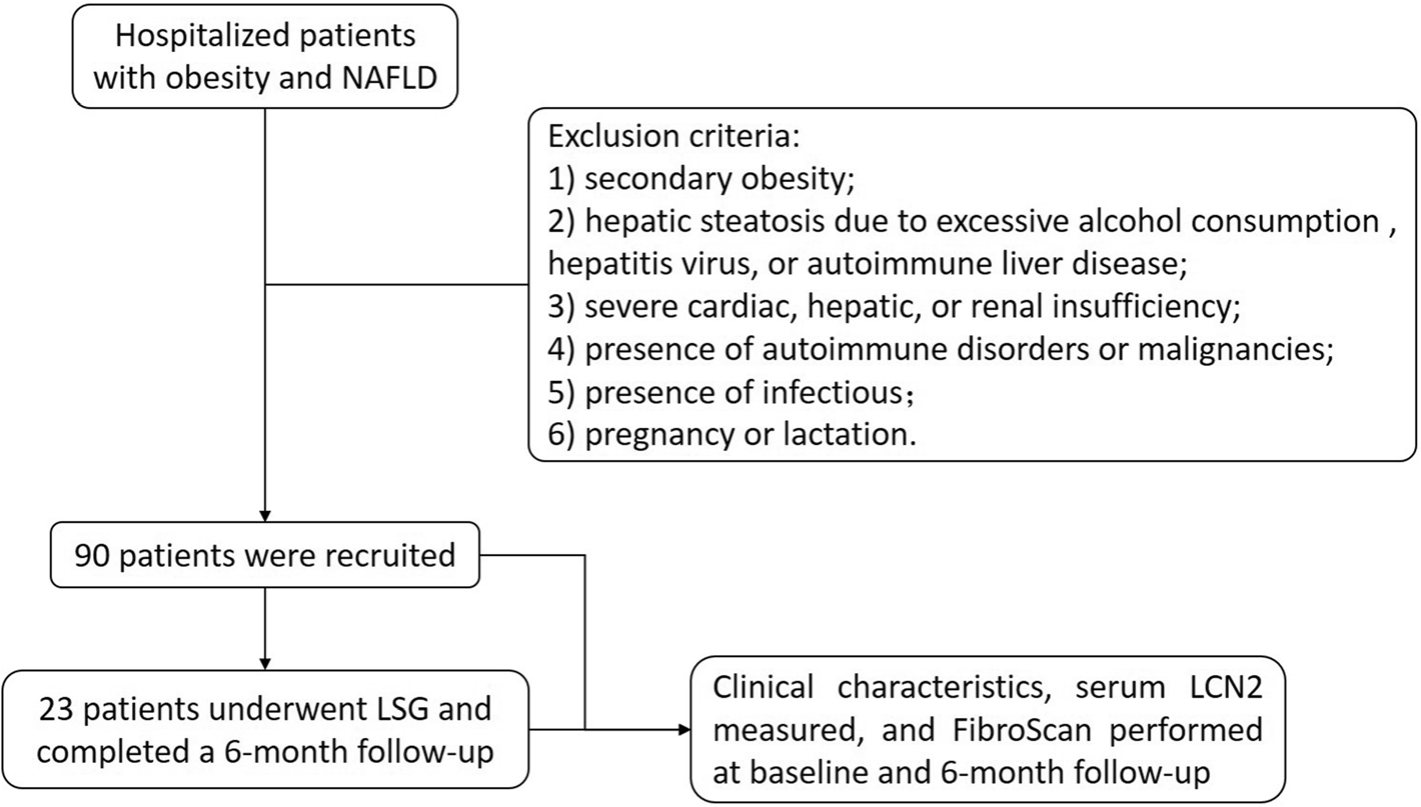
Study flow chart

The present work is a retrospective observational study. Altogether 90 obese patients with NAFLD from the Department of Endocrinology and Metabolism, Shanghai Tenth People’s Hospital were enrolled. Patient inclusion criteria were (1) age between 18 and 65 years; (2) body mass index (BMI) greater than 28 kg/m^2^; and (3) hepatic steatosis diagnosed with FibroScan. Patient exclusion criteria were (1) secondary obesity due to hypothalamic diseases, hypophysis dysfunction, thyroid disorders, gonadal diseases, Cushing syndrome, or genetic diseases; (2) hepatic steatosis due to excessive alcohol consumption (more than 210 g for men and more than 140 g for women per week), hepatitis virus, or autoimmune liver disease; 3) severe cardiac, hepatic, or renal insufficiency; 4) presence of autoimmune disorders or malignancies; 5) presence of infectious diseases; and 6)pregnancy or lactation. Among the included subjects, 23 of them underwent LSG and returned to hospital at 6 months after surgery for a comprehensive medical examination. Figure 1 shows the research flow chart. This work gained approval from the Ethics Committee of Shanghai Tenth People’s Hospital (NCT04573998). Each individual signed an informed consent for participation.

### Clinical and biochemical parameters

Anthropometric parameters were measured by professional staff. Venous blood was obtained after overnight fasting. Biochemical parameters including alanine aminotransferase (ALT), aspartate aminotransferase (AST), glutamyltransferase (γGT), fasting blood glucose, HbA1c, triglyceride (TG), and total cholesterol (TC) were then measured.

### Serum LCN2 measurement

Serum LCN2 levels were determined by ELISA kit (DLCN20, R&D Systems) following manufacturer’s protocols.

### Assessment of NAFLD

The current study performed FibroScan, a noninvasive ultrasound-based method for evaluating hepatic steatosis. Controlled attenuation parameter (CAP), calculated from ultrasonic signals, is used to quantify hepatic steatosis. According to the value of CAP, steatosis was categorized into 3 grades: S1 for mild steatosis (fat content over 10%), S2 for moderate steatosis (fat content over 33%), and S3 for severe steatosis (fat content over 66%), with the CAP cut-off values of 238, 259, and 292 dB/m, respectively. FibroScan was performed in all patients at baseline and patients underwent LSG at 6-month follow-up.

### Statistical analysis

SPSS 20.0 was adopted for data analysis. Continuous distribution data are presented as the means ± SD. Quantitative data of normal distribution were compared by student’s t test. Qualitative data were compared by χ2-test and Kruskal-Wallis test. Linear regression and Pearson’s correlation were utilized for analyzing the association of the change of CAP value with change of LCN2 levels. Further, logistic regression was applied in analyzing the relationship between the improvement in steatosis grade and the change in LCN2 level. *P* < 0.05 was considered as statistical significance.

## Results

### Hepatic steatosis was more severe in severe obesity

The clinical parameters of the included patients at baseline are summarized in Table 1. Obese patients were divided into two groups according to their BMI. Differences in gender or age were not significant between the two groups. Plasma fasting glucose and serum TG and TC were similar between the two groups. As expected, the CAP value was higher in patients with severe obesity, indicating more severe hepatic steatosis. However, the liver function of ALT, AST, and γGT between the two groups had no significant difference.

**Table 1:** Clinical characteristics of the patients

	all	BMI < 37.5	BMI ≥ 37.5	*P* value
n	90	37	53	/
Male [n(%)]	43 (47.8)	15 (40.5)	28 (52.8)	0.251
Age (years)	30.58 ± 9.48	31.78 ± 9.78	29.74 ± 9.26	0.316
Waist (cm)	119.98 ± 15.23	106.95 ± 11.23	128.56 ± 10.79	< 0.001
ALT (U/L)	58.94 ± 50.80	57.56 ± 55.43	59.90 ± 47.83	0.831
AST (U/L)	32.70 ± 23.72	29.82 ± 20.66	34.71 ± 25.63	0.339
γGT (U/L)	48.99 ± 39.28	40.35 ± 22.18	54.40 ± 46.34	0.113
Glucose (mmol/L)	5.97 ± 1.94	6.18 ± 2.21	5.82 ± 1.73	0.405
HbA1c (%)	6.78 ± 1.87	6.76 ± 2.09	6.80 ± 1.72	0.919
TG (mmol/L)	1.91 ± 1.08	2.03 ± 0.98	1.83 ± 1.15	0.400
TC (mmol/L)	4.60 ± 0.80	4.61 ± 0.86	4.59 ± 0.77	0.899
LCN2 (ng/mL)	103.81 ± 45.26	92.68 ± 32.68	111.59 ± 51.16	0.035
CAP (dB/m)	352.40 ± 45.88	340.78 ± 45.02	360.51 ± 45.14	0.044

Results are shown as mean ± SD or number (percentage)

BMI: body mass index; ALT: alanine aminotransferase; AST: aspartate aminotransferase; γGT: glutamyltransferase; TG: triglyceride; TC: total cholesterol; CAP: controlled attenuation parameter

### Metabolic parameters were improved after LSG

Among the included patients, 23 underwent LSG and completed a follow-up after six months. Body weight and waist circumstance were significantly decreased. Fasting glucose, HbA1c, TG, and TC also showed a significant decrease, indicating an improvement of glucose metabolism and lipid metabolism accompanied by weight loss after LSG (Table 2).

**Table 2:** Clinical characteristics after LSG

	baseline	post-surgery	*P* value
Weight (kg)	111.13 ± 27.92	85.81 ± 24.99	< 0.001
BMI (kg/m2)	38.45 ± 7.55	29.56 ± 6.54	< 0.001
Waist (cm)	122.09 ± 15.91	101.41 ± 13.32	< 0.001
ALT (U/L)	78.38 ± 75.09	15.22 ± 8.61	0.001
AST (U/L)	40.91 ± 28.55	16.59 ± 5.80	< 0.001
γGT (U/L)	43.34 ± 23.85	14.56 ± 8.50	< 0.001
Glucose (mmol/L)	5.98 ± 1.48	4.45 ± 0.54	< 0.001
HbA1c (%)	7.16 ± 2.35	5.43 ± 0.35	0.002
TG (mmol/L)	2.02±0.98	1.05 ± 0.45	< 0.001
TC (mmol/L)	4.58 ± 0.96	4.07 ± 0.72	0.003
LCN2 (ng/mL)	119.74 ± 36.15	87.38 ± 51.65	0.001
CAP (dB/m)	358.48 ± 46.13	260.83 ± 69.64	< 0.001
Steatosis grade [n(%)]			< 0.001
S0	0 (0)	8 (34.8)	
S1	2 (8.7)	5 (21.7)	
S2	0 (0)	2 (8.7)	
S3	21 (91.3)	8 (34.8)	

Results are shown as mean ± SD or number (percentage)

BMI: body mass index; ALT: alanine aminotransferase; AST: aspartate aminotransferase; γGT: glutamyltransferase; TG: triglyceride; TC: total cholesterol; CAP: controlled attenuation parameter

### Hepatic steatosis was improved after LSG

In addition to metabolic parameters, liver function was also improved suggested by significant decreases in serum levels of ALT, AST, and γGT. FibroScan was also performed to reevaluate hepatic steatosis. The CAP value decreased from 358.48 ± 46.13 dB/m to 260.83 ± 69.64 dB/m (*P* < 0.001). Steatosis grade was also improved after LSG (Table 2).

### Serum LCN2 levels increased in severe obesity and decreased after LSG

For patients with severe obesity, serum LCN2 levels were much higher (Table 1), suggesting that LCN2 levels were elevated in individuals with higher BMI and more severe hepatic steatosis. In the subgroup of patients who underwent LSG, serum LCN2 levels decreased from 119.74 ± 36.15 ng/mL to 87.38 ± 51.65 ng/mL (*P* < 0.001) six months after surgery (Table 2).

### Change in serum LCN2 levels was related to hepatic steatosis improvement

The authors performed Pearson’s correlation (Table 3) for investigating the relationship between decrease in LCN2 levels and hepatic steatosis improvement. The decreased CAP value showed positive association with a decrease in the LCN2 levels (r = 0.432, *P* = 0.040). For further exploring whether the change of serum LCN2 level contributed to the change of CAP value, the authors conducted linear regression analysis (Table 4). The change of CAP value showed positive association with the change of LCN2 levels after age, gender, and change in BMI were adjusted. As revealed by logistic regression, improvement in steatosis grade was also correlated with LCN2 decrease (Table 5).

**Table 3:** Associations between change in CAP value and change in LCN2 level and liver function

	ΔCAP	
	r	*P*
ΔBMI	0.029	0.896
ΔALT	0.411	0.051
ΔAST	0.372	0.080
ΔγGT	0.394	0.106
ΔLCN2	0.432	0.040

BMI: body mass index; ALT: alanine aminotransferase; AST: aspartate aminotransferase; γGT: glutamyltransferase; CAP: controlled attenuation parameter

**Table 4:** Multivariate regression regarding the association of decrease in CAP value and decrease in LCN2 level

model	ΔCAP		
	Β	R square	*P* value
1	0.666	0.186	0.040
2	0.776	0.218	0.037
3	0.765	0.218	0.049

Model 1: ΔLCN2; model 2: ΔLCN2 after age and gender were adjusted; model 3: ΔLCN2 after age, gender, and ΔBMI were adjusted

CAP: controlled attenuation parameter

**Table 5:** Logistic regression analysis of factors associated with steatosis grade improvement

	OR	95%CI	*P* value
Gender	3.746	0.333 - 42.144	0.285
Age (years)	0.941	0.842 - 1.051	0.279
ΔBMI (kg/m2)	0.964	0.611 - 1.522	0.876
ΔLCN2 (ng/mL)	1.044	1.004 - 1.085	0.031

BMI: body mass index

## Discussion

Weight loss is accompanied by metabolic improvement following bariatric surgery, which has been now considered an effective approach to NAFLD. For both retrospective and prospective cohort studies, NAFLD was improved after bariatric surgery after evaluating serum biomarkers, hepatic steatosis, inflammation, and fibrosis [10, 11]. In this study, the authors used the CAP value calculated by FibroScan to evaluate hepatic steatosis. It is of great importance to quantify hepatic steatosis for evaluating NAFLD. However, the gold standard, namely, liver biopsy, has not been extensively adopted in clinical practice because it is an invasive measurement. Reliable noninvasive methods are thus needed for replacement. Serum biomarkers include predictive models such as the Hepatic Steatosis Index and Fatty Liver Index. These scores have been validated by separate studies. However, they have not gained much popularity in clinical practice because these models cannot provide additional information for patients with NAFLD [12]. FibroScan is an ultrasound-based method that is widely used in clinical practice. It has been proven to be accurate and convenient in evaluating hepatic steatosis in different populations [13, 14]. The present study also revealed that liver function and hepatic steatosis (CAP value and steatosis grade) were significantly improved after bariatric surgery. And circulating LCN2 levels were declined in parallel among patients after LSG.

As mentioned above, LCN2 was recently found to be associated with obesity and its related metabolic comorbidities. Circulating LCN2 increased among obese patients compared with normal body weight individuals. For obese individuals, up-regulation of LCN2 was also observed in adipose tissue [15]. Similar results were confirmed by other human studies and animal studies. LCN2 expression was then investigated in obesity related metabolic disorders. However, regarding NAFLD, the results from different studies are inconsistent [16].

Several studies have indicated that LCN2 levels in circulation are increased among NAFLD patients. Other researchers found no differences between patients with NAFLD and healthy controls and doubted that the elevated LCN2 level was due to a higher BMI in patients with NAFLD [17]. A study conducted by Auguet T and colleagues [9] investigated LCN2 expression in a group of severely obese women to exclude the influence of gender, age, and BMI. They discovered that circulating LCN2 levels were up-regulated among morbidly obese women with NAFLD compared with those with normal liver. So were the hepatic LCN2 protein and mRNA levels. A recent study [18] included 360 patients with NAFLD and 40 healthy controls and discovered that circulating LCN2 levels were increased in NAFLD patients. Furthermore, the authors found that LCN2 level had a positive association with hepatic steatosis. Results of unchanged LCN2 level from some studies may be due to small number of patients enrolled and population selection. For most studies, LCN2 levels were elevated in NAFLD and found to be correlated with hepatic steatosis. In the current study, the authors demonstrated that serum LCN2 levels markedly declined after bariatric surgery. The decrease in LCN2 was positively correlated with the hepatic steatosis improvement after age, gender, and the decrease in BMI were adjusted. Together with former studies, the present study suggested that circulating LCN2 could be a biomarker for NAFLD.

Upregulation of LCN2 levels may have hepatoprotective effects in NAFLD [19]. A study conducted by E. Borkham-Kamphorst and colleagues [20] found that, after exposure to acute or chronic stimuli, increased liver damage and inflammatory cytokine expression were shown in LCN2^−/−^ mice. In LCN2^−/−^ mice, hepatocytes also exhibited more lipid drop deposition and increased cell apoptosis. H. Guo and colleagues [21] also suggested that LCN2^−/−^ mice were more susceptible to fatty liver caused by high-fat diet (HFD) because of enhanced hepatic insulin resistance as well as impaired lipid metabolism. Another study [22] used HFD and methionine-/choline-deficient diet for inducing hepatic steatosis and steatohepatitis and found that LCN2^−/−^ mice accumulated more hepatic lipids in both two models. The same study revealed that LCN2 modulated hepatic lipid homeostasis by regulating lipid droplet coat protein Perilipin 5 expression. Hepatic damage and steatosis were also reported to be prominent in LCN2^−/−^ mice under high-fructose diet by Lambertz and colleagues [23]. According to their results, LCN2 participated in the hepatic lipid uptake in a direct or indirect manner. Recently, XU and colleagues [24] found that LCN2 overexpression within mouse hepatocytes protected from diet-induced liver steatosis, and LCN2-deficient mice presented the opposite phenotype. Their results also revealed that LCN2 protected against diet-induced NAFLD through suppressing lipogenesis and promoting lipid oxidation and lipolysis. In addition to lipid metabolism, LCN2 was reported to regulate mitochondrial integrity and endoplasmic reticulum stress in hepatocytes [25], which are also involved in the pathophysiology of NAFLD.

## Strength and limitations

Previous studies revealed that circulating LCN2 levels increased in NAFLD. In addition to its elevation in NAFLD, the current study also demonstrated that LCN2 levels were correlated with steatosis grade and that the decrease in circulating LCN2 levels was correlated with the improvement in hepatic steatosis after bariatric surgery. However, some limitations should be noted in the current study. Firstly, this work was conducted at a single center, and the size of the subgroup of bariatric surgery patients was small. Secondly, CAP is not the most accurate method to assess hepatic steatosis compared to liver biopsy. Finally, this study only showed an association between circulating LCN2 levels and hepatic steatosis, the causal relationship was not explained. Thus, more studies are warranted to reveal the mechanism by which LCN2 participates in the improvement of NAFLD.

## Conclusions

In conclusion, this study revealed that circulating LCN2 levels were increased among patients with higher BMI and more severe hepatic steatosis. After bariatric surgery, the LCN2 level was significantly decreased together with the improvement of NAFLD. A decrease in LCN2 levels and a decrease in liver fat content were positively correlated. Circulating LCN2 levels may be a biomarker for hepatic steatosis severity and be monitored during the follow-up of NAFLD treatment in the future.

## Data Availability

All data in this study can be obtained from the corresponding author upon request.

## Abbreviations

ALT: alanine aminotransferase
AST: aspartate aminotransferase
BMI: body mass index
CAP: controlled attenuation parameter
γGT: glutamyltransferase
HFD: high-fat diet
LCN2: Lipocalin-2
LSG: laparoscopic sleeve gastrectomy
NAFLD: nonalcoholic fatty liver disease
TC: total cholesterol
TG: triglyceride

## References

[1] Diehl AM, Day C (2017). Cause, Pathogenesis, and Treatment of Nonalcoholic Steatohepatitis. N Engl J Med.

[2] Younossi Z, Tacke F, Arrese M, Chander Sharma B, Mostafa I, Bugianesi E, Wai-Sun Wong V, Yilmaz Y, George J, Fan J, Vos MB (2019). Global Perspectives on Nonalcoholic Fatty Liver Disease and Nonalcoholic Steatohepatitis. Hepatology.

[3] Younossi Z, Anstee QM, Marietti M, Hardy T, Henry L, Eslam M, George J, Bugianesi E (2018). Global burden of NAFLD and NASH: trends, predictions, risk factors and prevention. Nat Rev Gastroenterol Hepatol.

[4] Polyzos SA, Kountouras J, Mantzoros CS (2019). Obesity and nonalcoholic fatty liver disease: From pathophysiology to therapeutics. Metabolism.

[5] Fakhry TK, Mhaskar R, Schwitalla T, Muradova E, Gonzalvo JP, Murr MM (2019). Bariatric surgery improves nonalcoholic fatty liver disease: a contemporary systematic review and meta-analysis. Surg Obes Relat Dis.

[6] Goetz DH, Holmes MA, Borregaard N, Bluhm ME, Raymond KN, Strong RK (2002). The neutrophil lipocalin NGAL is a bacteriostatic agent that interferes with siderophore-mediated iron acquisition. Mol Cell.

[7] Yan QW, Yang Q, Mody N, Graham TE, Hsu CH, Xu Z, Houstis NE, Kahn BB, Rosen ED (2007). The adipokine lipocalin 2 is regulated by obesity and promotes insulin resistance. Diabetes.

[8] Meier EM, Pohl R, Rein-Fischboeck L, Schacherer D, Eisinger K, Wiest R, Krautbauer S, Buechler C (2016). Circulating lipocalin 2 is neither related to liver steatosis in patients with non-alcoholic fatty liver disease nor to residual liver function in cirrhosis. Cytokine.

[9] Auguet T, Terra X, Quintero Y, Martinez S, Manresa N, Porras JA, Aguilar C, Orellana-Gavalda JM, Hernandez M, Sabench F (2013). Liver lipocalin 2 expression in severely obese women with non alcoholic fatty liver disease. Exp Clin Endocrinol Diabetes.

[10] Chaim FDM, Pascoal LB, Chaim FHM, Palma BB, Damazio TA, da Costa LBE, Carvalho R, Cazzo E, Gestic MA, Utrini MP (2020). Histological grading evaluation of non-alcoholic fatty liver disease after bariatric surgery: a retrospective and longitudinal observational cohort study. Sci Rep.

[11] Lassailly G, Caiazzo R, Ntandja-Wandji LC, Gnemmi V, Baud G, Verkindt H, Ningarhari M, Louvet A, Leteurtre E, Raverdy V (2020). Bariatric Surgery Provides Long-term Resolution of Nonalcoholic Steatohepatitis and Regression of Fibrosis. Gastroenterology.

[12] Castera L, Friedrich-Rust M, Loomba R (2019). Noninvasive Assessment of Liver Disease in Patients With Nonalcoholic Fatty Liver Disease. Gastroenterology.

[13] Eddowes PJ, Sasso M, Allison M, Tsochatzis E, Anstee QM, Sheridan D, Guha IN, Cobbold JF, Deeks JJ, Paradis V (2019). Accuracy of FibroScan Controlled Attenuation Parameter and Liver Stiffness Measurement in Assessing Steatosis and Fibrosis in Patients With Nonalcoholic Fatty Liver Disease. Gastroenterology.

[14] Naveau S, Voican CS, Lebrun A, Gaillard M, Lamouri K, Njike-Nakseu M, Courie R, Tranchart H, Balian A, Prevot S (2017). Controlled attenuation parameter for diagnosing steatosis in bariatric surgery candidates with suspected nonalcoholic fatty liver disease. Eur J Gastroenterol Hepatol.

[15] Catalan V, Gomez-Ambrosi J, Rodriguez A, Ramirez B, Silva C, Rotellar F, Gil MJ, Cienfuegos JA, Salvador J, Fruhbeck G (2009). Increased adipose tissue expression of lipocalin-2 in obesity is related to inflammation and matrix metalloproteinase-2 and metalloproteinase-9 activities in humans. J Mol Med (Berl).

[16] Krizanac M, Mass Sanchez PB, Weiskirchen R, Asimakopoulos A (2021). A Scoping Review on Lipocalin-2 and Its Role in Non-Alcoholic Steatohepatitis and Hepatocellular Carcinoma. Int J Mol Sci.

[17] Singh RG, Nguyen NN, Cervantes A, Kim JU, Stuart CE, Petrov MS (2019). Circulating levels of lipocalin-2 are associated with fatty pancreas but not fatty liver. Peptides.

[18] Xu G, Wang YM, Ying MM, Chen SD, Li ZR, Ma HL, Zheng MH, Wu J, Ding C (2021). Serum lipocalin-2 is a potential biomarker for the clinical diagnosis of nonalcoholic steatohepatitis. Clin Mol Hepatol.

[19] Asimakopoulou A, Weiskirchen S, Weiskirchen R (2016). Lipocalin 2 (LCN2) Expression in Hepatic Malfunction and Therapy. Front Physiol.

[20] Borkham-Kamphorst E, van de Leur E, Zimmermann HW, Karlmark KR, Tihaa L, Haas U, Tacke F, Berger T, Mak TW, Weiskirchen R (2013). Protective effects of lipocalin-2 (LCN2) in acute liver injury suggest a novel function in liver homeostasis. Biochim Biophys Acta.

[21] Guo H, Jin D, Zhang Y, Wright W, Bazuine M, Brockman DA, Bernlohr DA, Chen X (2010). Lipocalin-2 deficiency impairs thermogenesis and potentiates diet-induced insulin resistance in mice. Diabetes.

[22] Asimakopoulou A, Borkham-Kamphorst E, Henning M, Yagmur E, Gassler N, Liedtke C, Berger T, Mak TW, Weiskirchen R (2014). Lipocalin-2 (LCN2) regulates PLIN5 expression and intracellular lipid droplet formation in the liver. Biochim Biophys Acta.

[23] Lambertz J, Berger T, Mak TW, van Helden J, Weiskirchen R (2017). Lipocalin-2 in Fructose-Induced Fatty Liver Disease. Front Physiol.

[24] Xu Y, Zhu Y, Jadhav K, Li Y, Sun H, Yin L, Kasumov T, Chen X, Zhang Y (2019). Lipocalin-2 Protects Against Diet-Induced Nonalcoholic Fatty Liver Disease by Targeting Hepatocytes. Hepatol Commun.

[25] Asimakopoulou A, Fulop A, Borkham-Kamphorst E, de Leur EV, Gassler N, Berger T, Beine B, Meyer HE, Mak TW, Hopf C (2017). Altered mitochondrial and peroxisomal integrity in lipocalin-2-deficient mice with hepatic steatosis. Biochim Biophys Acta Mol Basis Dis.

