# Correction: The relationship between Lipocalin-2 level and hepatic steatosis in obese patients with NAFLD after bariatric surgery

**DOI:** 10.1186/s12944-023-01779-2

**Published:** 2023-02-27

**Authors:** Jiaqi Chen, Shihui Lei, Yueye Huang, Xiaojuan Zha, Lei Gu, Donglei Zhou, Jun Li, Feng Liu, Nannan Li, Lei Du, Xiu Huang, Ziwei Lin, Le Bu, Shen Qu

**Affiliations:** 1 Department of Endocrinology and Metabolism, Shanghai Tenth People’s Hospital, Clinical Medicine School of Nanjing Medical University, Medicine School of Tongji University, Shanghai, 200072 China; 2 Department of Endocrinology and Metabolism, Suzhou Municipal Hospital, The Affiliated Suzhou Hospital of Nanjing Medical University, Suzhou, China; 3 Department of Gastrointestinal Surgery, Shanghai Tenth People’s Hospital, Clinical Medicine School of Nanjing Medical University, Medicine School of Tongji University, Shanghai, 200072 China; 4 Department of Gastroenterology, Shanghai Tenth People’s Hospital, Clinical Medicine School of Nanjing Medical University, Medicine School of Tongji University, Shanghai, 200072 China

**Correction: Lipids Health Dis 21, 10 (2022)**

**https://doi.org/10.1186/s12944-022-01622-0**

Following publication of the original article [1], the authors reported an error in affiliations.

The accurate author affiliations should be as follows:

1. Department of Endocrinology and Metabolism, Shanghai Tenth People's Hospital, Clinical Medicine School of Nanjing Medical University, Medicine School of Tongji University, Shanghai 200072, China.

2. Department of Endocrinology and Metabolism, Suzhou Municipal Hospital, The Affiliated Suzhou Hospital of Nanjing Medical University, Suzhou, China.

3. Department of Gastrointestinal Surgery, Shanghai Tenth People's Hospital, Clinical Medicine School of Nanjing Medical University, Medicine School of Tongji University, Shanghai 200072, China.

4. Department of Gastroenterology, Shanghai Tenth People's Hospital, Clinical Medicine School of Nanjing Medical University, Medicine School of Tongji University, Shanghai 200072, China.

The original article has been updated.
